# Investigation of Thermal Stability of Microstructure and Mechanical Properties of Bimetallic Fine-Grained Wires from Al–0.25%Zr–(Sc,Hf) Alloys

**DOI:** 10.3390/ma15010185

**Published:** 2021-12-27

**Authors:** Aleksey Nokhrin, Iana Shadrina, Vladimir Chuvil’deev, Vladimir Kopylov, Nikolay Berendeev, Artem Murashov, Aleksandr Bobrov, Nataliya Tabachkova, Elena Smirnova, Mikhail Faddeev

**Affiliations:** 1 Materials Science Department, Physical-Technical Research Institute, Lobachevsky State University of Nizhniy Novgorod, 603022 Nizhny Novgorod, Russia; janashadr@gmail.com (I.S.); chuvildeev@nifti.unn.ru (V.C.); kopylov.ecap@nifti.unn.ru (V.K.); ear13@mail.ru (N.B.); aamurashov@nifti.unn.ru (A.M.); aabobrov@bk.ru (A.B.); smirnova@nifti.unn.ru (E.S.); faddeev@yandex.ru (M.F.); 2 Laboratory of Vacuum Plasma Coating, Physical-Technical Institute, National Academy of Sciences of Belarus, 220141 Minsk, Belarus; 3 Center Collective Use “Materials Science and Metallurgy”, National University of Science and Technology “MISIS”, 119991 Moscow, Russia; ntabachkova@misis.ru; 4 Laboratory “FIANIT”, Laser Materials and Technology Research Center, A. M. Prokhorov General Physics Institute, Russian Academy of Sciences, 119991 Moscow, Russia

**Keywords:** aluminum alloys, microalloying, microhardness, electrical conductivity, zirconium, scandium, hafnium

## Abstract

Thermal stability of composite bimetallic wires from five novel microalloyed aluminum alloys with different contents of alloying elements (Zr, Sc, and Hf) is investigated. The alloy workpieces were obtained by induction-casting in a vacuum, preliminary severe plastic deformation, and annealing providing the formation of a uniform microstructure and the nucleation of stabilizing intermetallide Al_3_(Zr,Sc,Hf) nanoparticles. The wires of 0.26 mm in diameter were obtained by simultaneous deformation of the Al alloy with Cu shell. The bimetallic wires demonstrated high strength and improved thermal stability. After annealing at 450–500 °C, a uniform fine-grained microstructure formed in the wire (the mean grain sizes in the annealed Al wires are 3–5 μm). An increased hardness and strength due to nucleation of the Al_3_(Sc,Hf) particles was observed. A diffusion of Cu from the shell into the surface layers of the Al wire was observed when heating up to 400–450 °C. The Cu diffusion depth into the annealed Al wire surfaces reached 30–40 μm. The maximum elongation to failure of the wires (20–30%) was achieved after annealing at 350 °C. The maximum values of microhardness (H_v_ = 500–520 MPa) and of ultimate strength (σ_b_ = 195–235 MPa) after annealing at 500 °C were observed for the wires made from the Al alloys alloyed with 0.05–0.1% Sc.

## 1. Introduction

The replacement of copper wires by aluminum ones is one of the relevant problems of modern aviation electrical engineering [1,2,3]. This would allow reducing the weight of the on-board electrical wiring of modern aircrafts at the same as increasing thermal stability, high electrical conductivity, and strength at elevated temperatures [4,5].

At present, complex Al alloys are the most promising material for the bimetallic wires of the types 55A, 55M, 55PC0811 (Spec-55), EN 2714-013A, etc. with Cu or Ag coatings. Complex Al-Ce-La-Fe Russian industrial alloy 01,417 is an example of such materials [4] as well as a number of novel conductor alloys that are doped with rare earth elements (REE) [6,7,8,9,10,11,12]. The 01,417 alloy has the minimal ultimate strength of 160 MPa, relative elongation to failure 8%, and specific electrical resistivity (SER) of 3.2 μΩ·cm [4]. Al wires of 0.2–0.3 mm in diameter with Cu or Ni coatings are being fabricated from this alloy [4]. Note that the wires that are made from 01,417 [4] и alloy of the known conductor Al-Mg-Si alloy family [1,5,13,14] don’t have the necessary thermal stability to allow replacing small-sized Cu wires efficiently. The requirements of the thermal stability of the structure and properties during long-term (several thousand hours) operation at 200–250 °C are imposed on modern aviation wires. In this connection, the novel conductor dispersion-hardened Al alloys are being developed intensively. The improved strength and thermal stability in these alloys are provided by the nucleation of Al_3_X nanoparticles where X = Sc, Zr, Er, and other alloying elements [15,16,17,18,19,20,21,22,23,24,25]. The alloying of the Al alloys by Sc is the most efficient [16,21,24,25]. It allows the ensuring of the high strength and thermal stability of the microstructure of the Al alloys. Sc is one of the most expensive alloying elements for the Al alloys. Therefore, the replacement of Sc by cheaper transition metals and/or REEs (Zr, Er, Hf, etc.) is an important practical task [15,16,17,18,19,20,21,22,23,24,25].

At present, the conductor Al-Zr alloys are being developed intensively [26,27,28,29]. In spite of prospects of the Al-Zr system in electrical engineering applications, the low nucleation intensity of the Al_3_Zr particles is a drawback of these alloys. The intensive nucleation of these particles takes place at high temperatures only (over 400–450 °C) and isothermic holding times (over 50 h) [26,27,28,29,30]. Note also that the decomposition of solid solutions in the Al-Zr and Al-Sc alloys may go via an intermittent precipitation mechanism with formation of large enough particles [21,30]. The complexity of these factors doesn’t allow stabilizing the ultrafine-grained (UFG) microstructure in Al since the grain boundaries in Al begin to migrate at 200–250 °C [31].

To solve this problem, two main approaches are applied at present. The first approach is the development of alloys with improved Zr content (0.4–0.6%Zr [26,27,28,29]) where the Zr concentration is much higher than the solubility limit of Zr in Al with the simultaneous application of ultrafast crystallization technology. This approach is hardly applicable for making the wires with small diameters since the primary large Al_3_Zr particles may lead to the wire breaking during drawing at room temperature or while rolling. The second approach is the development of Al-Zr alloys that are doped additionally by a third element affecting the Al-Zr alloy precipitation intensity. According to [5,16,24,25,32,33,34], the additional alloying of Al-Zr alloy by some rare elements (REE) or transition metals (Sc, Hf, Yb, Er, etc.) would lead to the accelerated nucleation of Al_3_Zr intermetallide nanoparticles, which should ensure the stabilization of the nonequilibrium UFG microstructure.

The present study was aimed at the fabrication and investigation of the thermal stability of the small-sized composite wires that were fabricated from novel dispersion-hardened microalloyed Al alloys; the Al-0.25%Zr alloys were the objects of investigation. The accelerated nucleation of the Al_3_Zr particles with the L1_2_ structure was provided by adding 0.05–0.1% of Sc and Hf.

## 2. Materials and Methods

**Figure 1:**
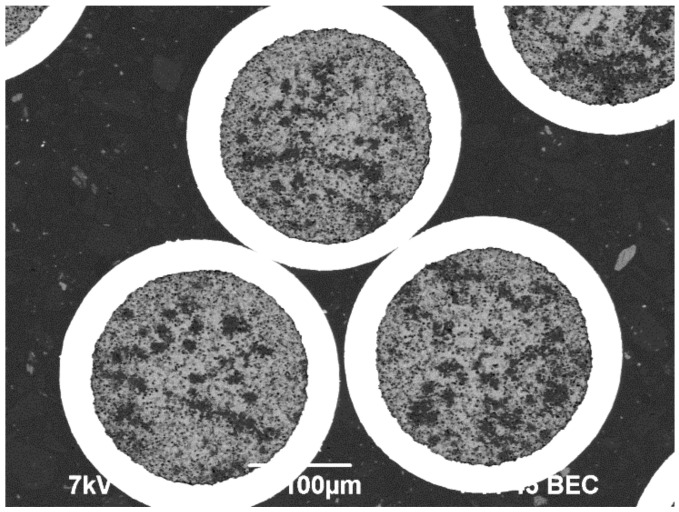
Cross-sections of the bimetallic wires. Scanning electron microscope (SEM).

The microalloyed Al alloys (Table 1) and the bimetallic Al wires of 0.26 mm in diameter that were obtained from these alloys with high-purity Cu coatings (~30 μm thick) were the objects of investigation. An example of cross-sections of the bimetallic wires is presented in Figure 1.

The wires made from pure Al A99 (Alloy #1) and Alloy #6 with a reduced Zr content (0.20%) and increased (0.1%) Sc while the Hf contents were used as the reference objects.

The Al workpieces of 22 × 22 mm^2^ in cross-sections were obtained by induction-casting from high-purity Al A99 using an INDUTHERM^®^ VTC-200 casting machine (Indutherm GmbH, Walzbachtal, Germany). The regimes of making the bulks are presented in Table 2. The uncertainty of the temperature measurements in the casting machine was 10 °C. The choice of the casting regimes was based on the analysis of our previous experiments on preparing the conductor Al alloys. In these regimes, a minimal quantity of the primary particles forms during crystallization and the cast alloy workpieces have the same macrostructure and close properties (microhardness, SER) in different bulk cross-sections. For alloying the alloys, Al-2%Zr, Al-2%Sc, and Al-2%Hf master alloys that were obtained by induction casting followed by rolling into foils of 0.2 mm in thickness were used. The foil that was obtained was cut into fragments of ~1 mm^2^ in size and were mixed with Al in preset proportions.

After casting, the Al alloy workpieces were subjected to equal channel angular pressing (ECAP) at 250 °C (the number of pressing cycles *n* = 4, mode B_c_) to remove the microstructure non uniformities. The selected ECAP temperature provided (i) the absence of micro- and macro-cracks in the Al alloy workpieces, (ii) forming a uniform fine-grained structure in the Al alloys; and (iii) the absence of nucleation of the Al_3_(TM,REE) particles in the course of ECAP. ECAP was performed using a Ficep^®^ HF 400 L hydraulic press (Ficep^®^ S.P.A., Varese, Italy). The workpieces that were obtained by ECAP were annealed in EKPS-10 air furnace (Smolensk SKTB SPU JSC, Smolensk, Russia) at 300–400 °C (2 h) to form a uniform recrystallized microstructure and for nucleating the Al_3_X particles providing the stability of the fine-grained structure of the alloys. The uncertainty of the temperature maintenance in the furnace was 10 °C. After annealing, the workpiece surfaces were scalped to remove the oxides. The sample bimetallic wires were obtained by rolling of the Al workpieces with Cu cladding in cylindrical rolls at room temperature. High-purity Cu (Russian industrial name M00k) was used as the cladding material.

The chemical analysis was performed using iCAP^®^ 6300-ICP-OES Radial View™ spectrometer with induction-coupled plasma (Thermo Scientific, Waltham, MA, USA). The microstructure investigations were performed using a Leica^®^ IM DRM metallographic optical microscope (Leica Microsystems GmbH, Wetzlar, Germany), Jeol^®^ JSM-6490 scanning electron microscope (SEM) (Jeol Ltd., Tokyo, Japan) that was equipped with Oxford Instruments^®^ INCA 350 energy dispersion spectrometer (EDS) (Oxford Instruments pls., Oxford, UK), and a Jeol^®^ JEM-2100 transmission electron microscope (TEM) (Jeol Ltd., Tokyo, Japan). For convenience of the microstructure investigations, the sample wires were pressed into WEM REM mixture (Cloeren Technology GmbH, Berlin, Germany) or into an epoxy cement; the polymerization temperature of which didn’t exceed 150 °C, the polymerization time was 10 min or less. Low polymerization temperatures and times allowed avoiding any effect of this process on the microstructure parameters and on the mechanical properties of the wires.

The microhardness H_v_ was measured in the centers of the wire cross-sections using a Qness^®^ A60+ microhardness tester (Qness GmbH, Golling, Austria) with the load of 20 g. The uncertainty of the hardness measurements was ±20 MPa. For the tear tests, Lloyd Instruments^®^ LR5K Plus universal breaking machine (Lloyd Instruments Ltd., West Sussex, UK) was used. The traverse travel speed was 10 mm/min. The 60 mm long sample wires were tested at room temperature according to Russian Standard GOST 10446–80 [35]. In the course of testing, “stress σ–strain ε” curves were recorded which the values of the ultimate strength σ_b_ and of relative elongation to failure δ were determined from. To obtain statistically reliable results, three samples or more were tested in each series. The mean uncertainty of the ultimate strength measurements was ±20 MPa. The fractographic investigations of the sample wire fractures after the tension tests were carried out using a TESCAN^®^ Vega™ II SEM (Tescan Orsay Holding, a.s., Brno, Czech Republic).

For the SER measurements by the eddy current method in the cast and fine-grained bulk alloys, a SIGMATEST^®^ 2.069 instrument (FOERSTER Int., Pittsburgh, PA, USA) was used. The uncertainty of the SER measurements was ±0.03 μΩ⋅cm. To measure the SER of the wires, an E7-8 digital L-C-R meter (KIP Etalon, Korolev, Russia) that allowed the measurement of the SER of wires with an uncertainty ±0.1 μΩ⋅cm was employed. The SER was measured on 0.6 m long sample wires. The cross-section areas of the wires were measured at three points with an uncertainty of 10 μm.

To study the thermal stability of the sample wires, they were subjected to 30 min annealing in ambient air using an EKPS-10 furnace. The uncertainty of the temperature maintenance was 10 °C.

## 3. Results

### 3.1. Investigation of the Alloys

The investigations that were performed have shown that the Sc concentration decreased during casting by 0.005–0.01 wt.% relative to the calculated one. The Zr and Hf concentrations matched the ones that were specified in Table 1. No oxide particle formation was observed by SEM methods.

**Figure 2:**
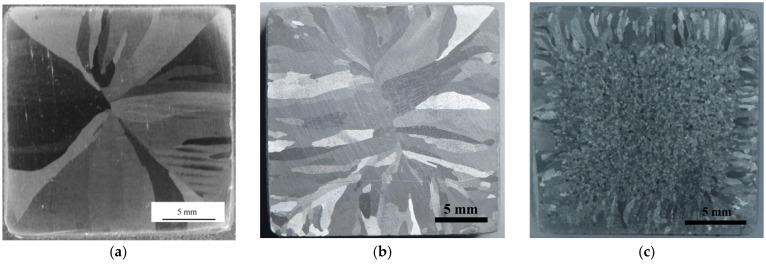

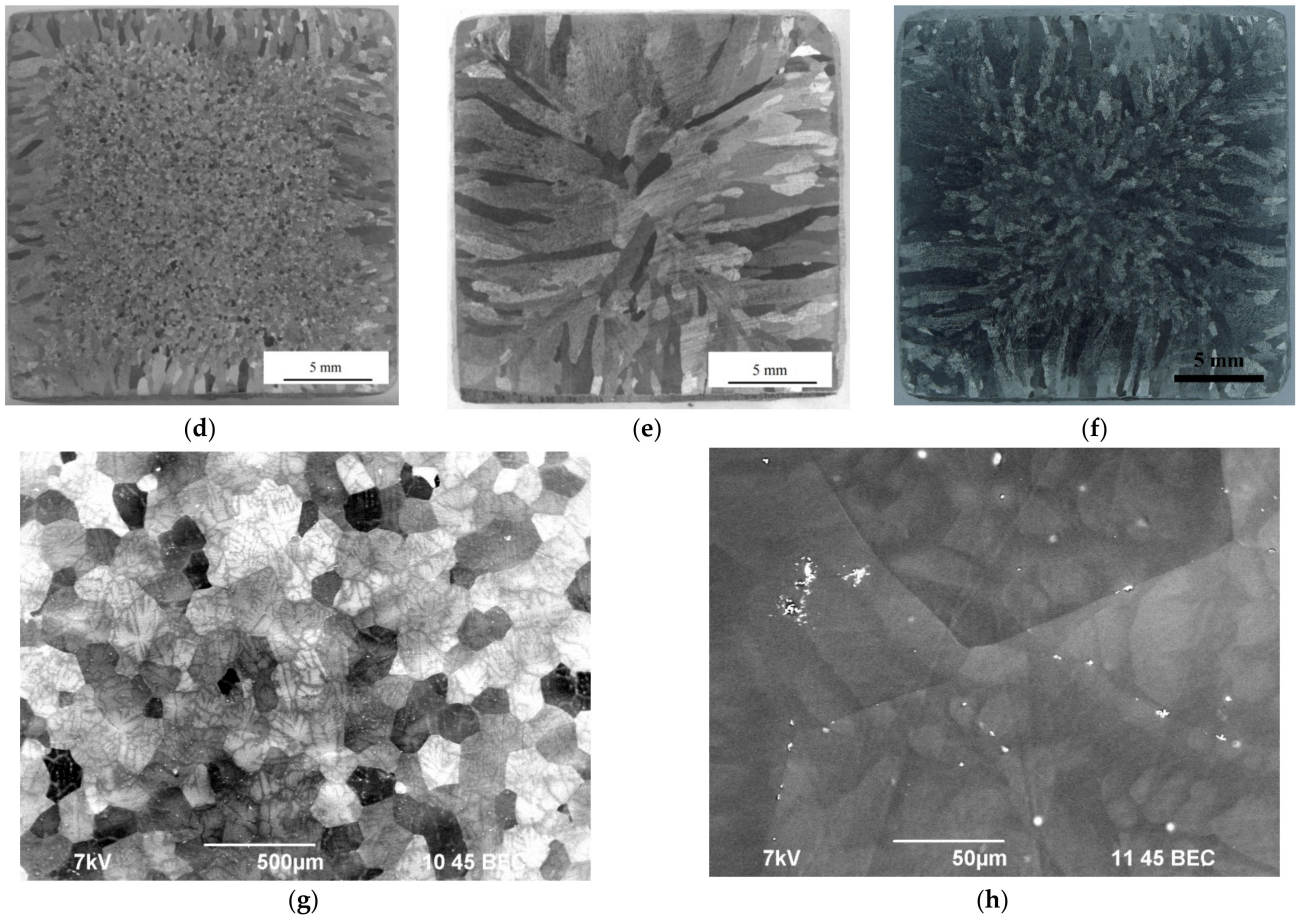
The microstructure of the cast alloys: (**a**) Alloy #1; (**b**) Alloy #2; (**c**) Alloy #3; (**d**) Alloy #4; (**e**,**g**,**h**) Alloy #5; and (**f**) Alloy #6. (**g**) The microstructure in the center of the bulk for Alloy #4; (**h**) the Al_3_Zr particles nucleated in the center of the bulk for Alloy #2.

The cast alloys in the initial state had a uniform coarse-grained structure consisting of columnar crystallites. At the sides of the cast bulks with the addition of 0.1%Sc (Alloy #3) or 0.1%Hf (Alloy #4), a columnar crystallite structure was observed compared with the central parts of the ones that consisted of uniaxial grains of ~280 μm in size (Figure 2c,d). The ratio of the areas that were occupied by each type of structure depended, first of all, on the type and concentration of the alloying elements. The macrostructures of Alloys #5 (Al-0.25%Zr-0.05%Sc-0.05%Hf) and #6 (Al-0.20%Zr-0.1%Sc-0.1%Hf) were the same and comprised large columnar crystals that were oriented from the bulk edges towards the centers. (Figure 2e,f) This means that the co-alloying with Sc and Hf were less efficient for the grinding of the cast Al macrostructure. In all the alloys, the primary Zr-containing particles were observed in the form of separate inclusions of ~1–3 μm in size as well as of aggregates of the micrometer-sized particles (Figure 2e).

The SER and microhardness of the alloys #1–6 in the initial state were 2.65 μΩ·cm and 200 MPa (Alloy #1), 2.87 μΩ·cm and 275 MPa (Alloy #2), 3.43 μΩ·cm and 350 MPa (Alloy #3), 3.36 μΩ·cm and 345 MPa (Alloy #4), 3.39 μΩ·cm and 315 MPa (Alloy #5), and 3.46 μΩ·cm and 340 MPa (Alloy #6), respectively.

**Figure 3:**
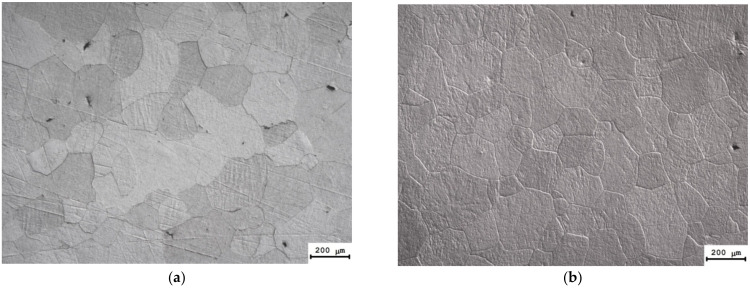
The microstructure of alloys after ECAP and annealing at 400 °C for 2 h: (**a**) Alloy #3; (**b**) Alloy #4. Metallography.

After ECAP, the Al alloy workpieces were subjected to 2 h annealing in the temperature range from 300 to 400 °C to ensure a uniformly recrystallized microstructure and to remove the dendrite nonuniformity (see Figure 2a–c), and to achieve the SER magnitude of the alloys 3.0 μΩ⋅cm or less. After annealing and 400 °C (2 h), a uniform microstructure was formed in the alloys #3 and 4 with the grain sizes ~150–200 μm (Figure 3a) and ~200 μm (Figure 3b), respectively. The mean grain sizes in alloys #5 and #6 containing Sc and Hf simultaneously were ~0.5–1 mm. The magnitudes of SER in the annealed alloys #2–6 were 2.92–2.95 μΩ⋅cm.

In alloys #1 and #2 (in which the magnitude of SER didn’t exceed 3.0 μΩ⋅cm) a uniform recrystallized microstructure formed after annealing at 300 °C (2 h). The mean grain sizes in the annealed alloys #1 and #2 were 0.5–1 mm, the magnitudes of SER were 2.66 and 2.84 μΩ⋅cm, respectively.

### 3.2. Investigation of the Wires

**Figure 4:**
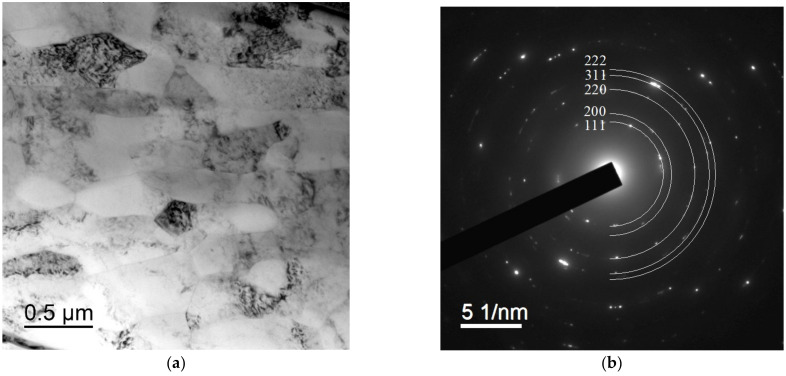

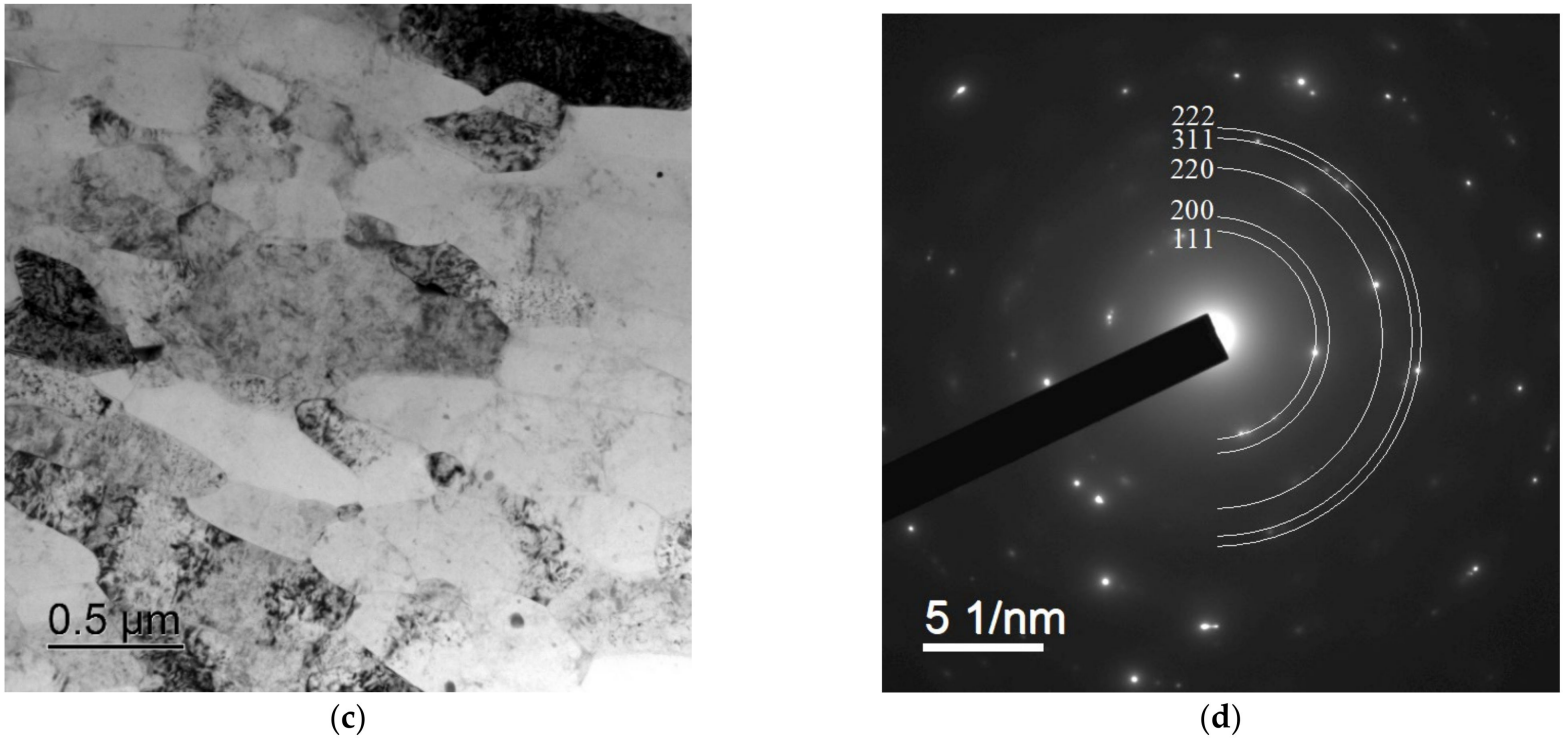
Microstructure of the central part of a bimetallic wire that was made from Alloy #2 (**a**,**b**) and Alloy #3 (**c**,**d**): (**a**,**c**) general view; (**b**,**d**) electron diffraction pattern. Transmission electron microscope (TEM).

**Figure 5:**
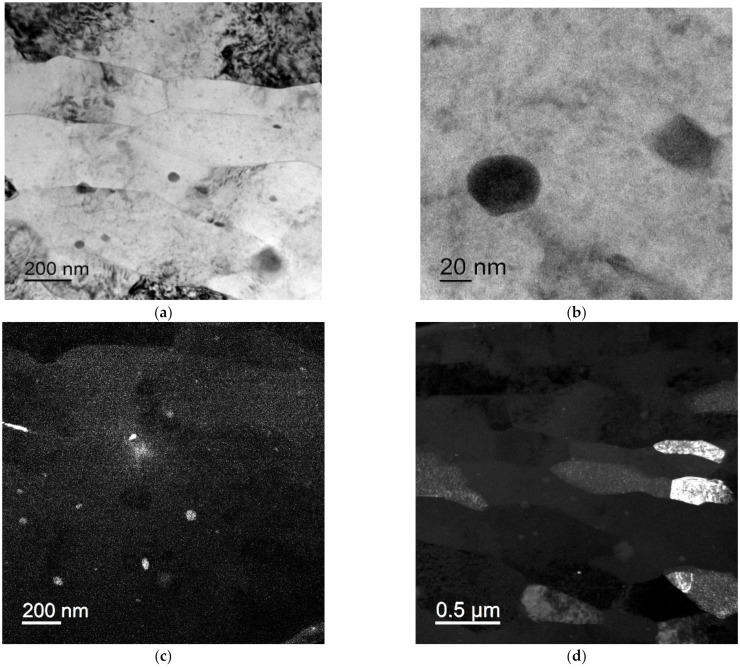

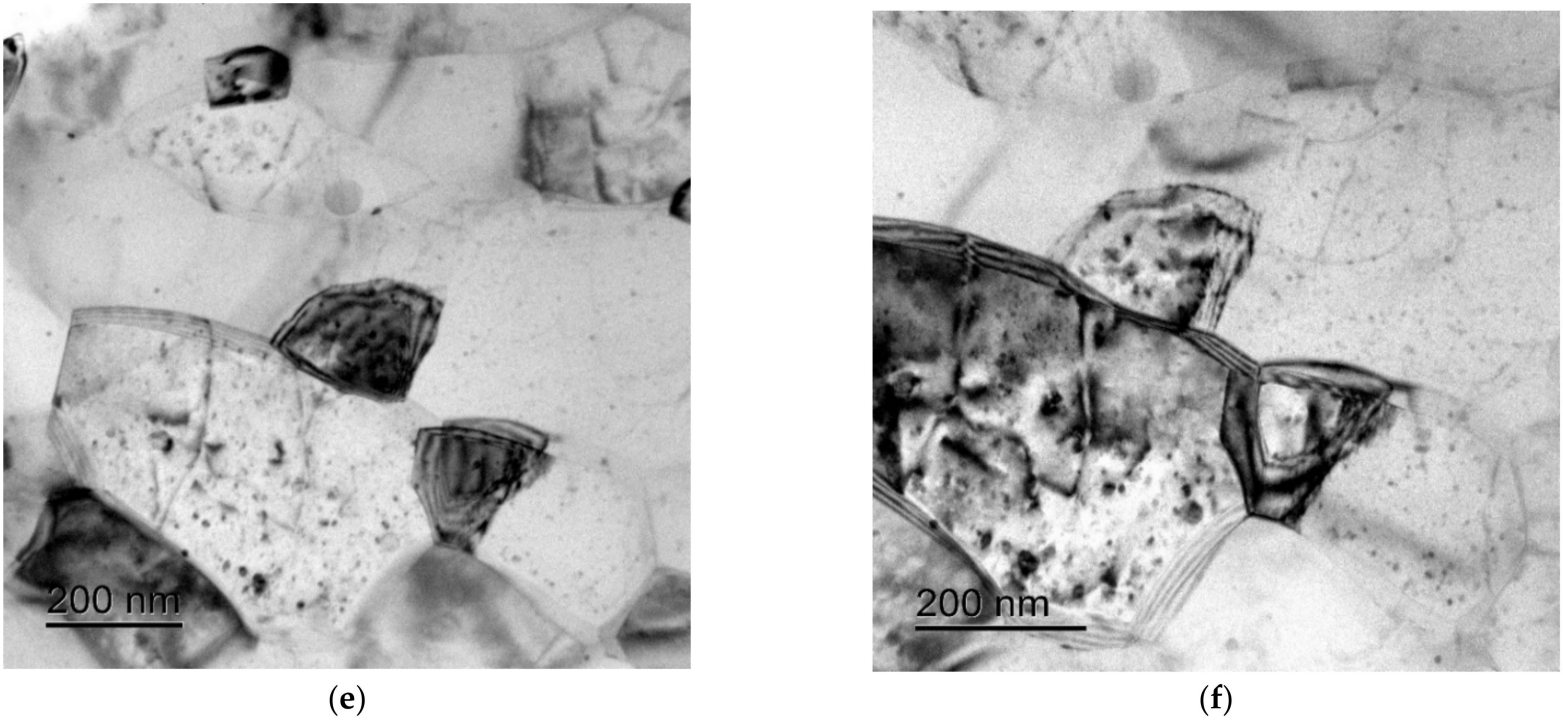
Al_3_(Zr,Sc) nanoparticles in the central part (**a**–**d**) and near the interphase boundary Al-Cu (**e**,**f**) of the sample bimetallic wire that was made from Alloy #3: (**a**,**b**) bright-field images; (**c**,**d**) dark-field images of different areas. TEM.

The bimetallic wires in the initial state had a strongly deformed mixed grained-subgrained microstructure with mean fragment sizes less than 0.5 μm (Figure 4). No essential differences in the microstructure of the wires that made from the alloys with different compositions were observed. There were the Al_3_(Sc,X) particles that were up to 20–30 nm in size inside the grains as well as at the grain boundaries in the Sc-containing alloys (Figure 5a,b). All the particles had regular, round shapes. No particle nucleation via intermittent decomposition was observed. Note that the selected particles were only observed in the dark field (Figure 5c,d). This was probably because the large particles were not coherent anymore so that the reflections from the ones were not manifested in the dark-field TEM images. No nanoparticle nucleation was observed in the wire that was made from Alloy #2 (Al-0.25%Zr) in the initial state.

**Figure 6:**
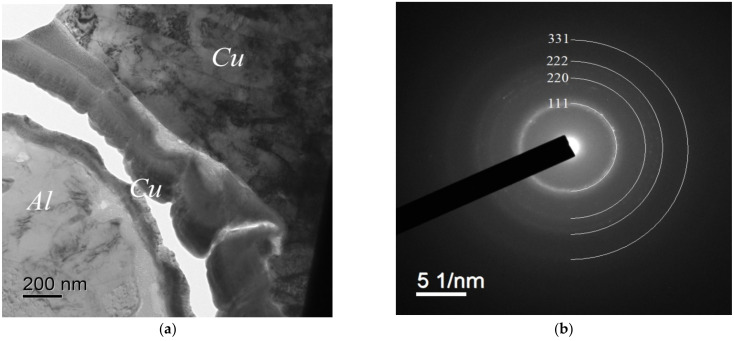

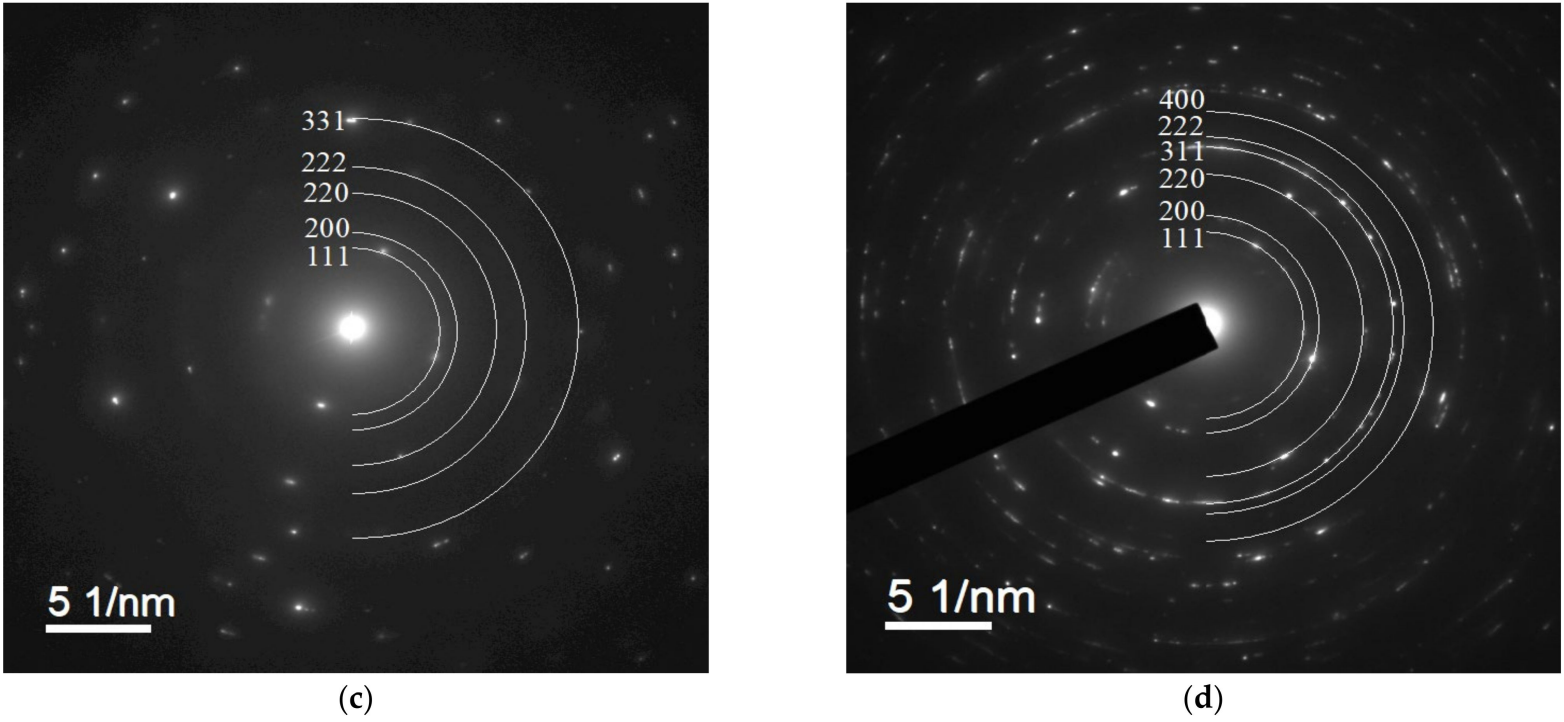
The results of TEM investigations of the interphase boundary “Al wire–Cu” in the initial state after rolling. Alloy #2: (**a**) general view of the investigated area (a crack formed that along the Al-Cu interphase boundary in the course of preparation of a thin foil for electron microscopy investigations is visible in Figure 6a. The presence of such cracks is an artifact of sample preparation. No interphase boundary destruction was found when investigating the polished samples by SEM). (**b**) The electron diffraction pattern of the interphase boundary Al–Cu; (**c**) electron diffraction pattern of the Al wire; (**d**) the same of the Cu cladding.

The investigations of the Cu cladding by electron microscopy demonstrated the amorphization of Cu to take place at the interface with an Al core as a result of severe plastic deformation; no characteristic reflections were observed in the electron diffraction pattern of the Al/Cu interphase boundary (Figure 6b) whereas all the reflections corresponding to the Al and Cu FCC phases were present in the electron diffraction patterns that were taken directly from the Al core (Figure 6c) and from the Cu cladding (Figure 6d), respectively.

**Figure 7:**
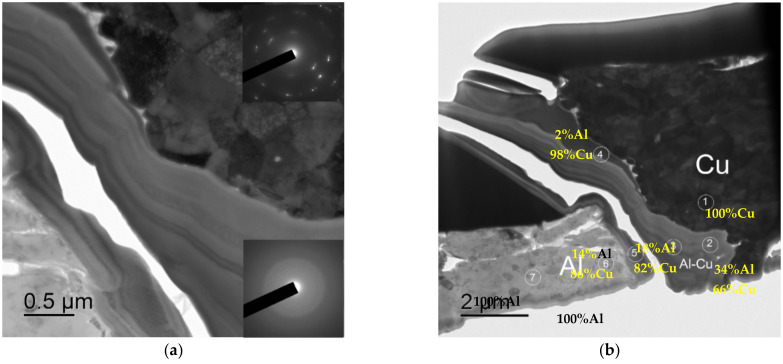
The results of the electron microscopy investigations of the interphase boundary in the bimetallic wire that were made from Alloy #3: (**a**) general view; (**b**) the results of the energy dispersion spectrometer (EDS) microanalysis (in wt.%).

One can see in Figure 6a and Figure 7a a strongly deformed fine-grained microstructure that formed in the Cu cladding. The mean sizes of the fragments were 0.2–0.5 μm.

According to the results of the EDS microanalysis, Cu and Al are present simultaneously in the amorphous phase at the Al-Cu interphase boundary (Figure 7). The Cu content in the Al matrix at the distances greater than 2–3 μm from the wire surface was small and didn’t exceed the measurement uncertainty.

**Figure 8:**
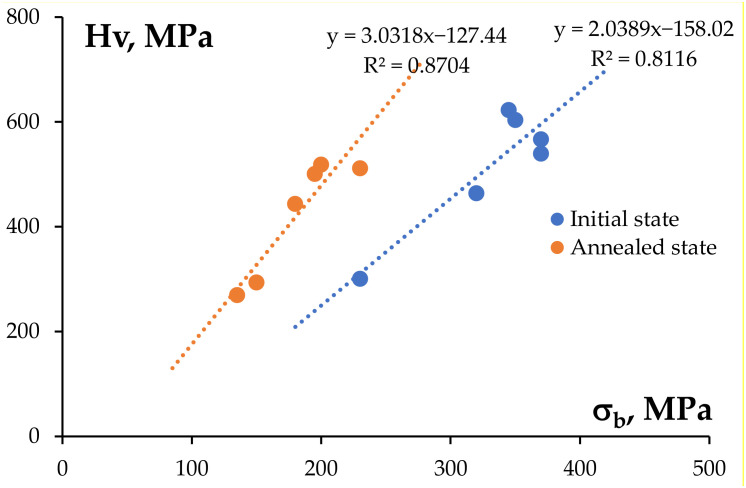
The dependencies H_V_(σ_b_) for the bimetallic wires in the non-annealed state and after annealing at 450 °C (30 min).

The results of investigations of the mechanical properties of the Al wires in the initial state are presented in Table 1. Comparing the results of the microhardness investigations of the bimetallic wires (Table 1) and of the cast alloys (see Section 3.1) demonstrated the severe plastic deformation of the Al workpiece by rolling that resulted in an increase in the hardness by a factor of 1.5–2. The minimum microhardness was observed for the wire made from pure Al (220 MPa) whereas the maximum microhardness was 605–625 MPa for the wires that were made from the alloys #3 and #6 containing 0.1%Sc. The microhardness values of the wires that were made from the Hf-containing alloys #4 and #5 were 540–565 MPa. These values are slightly lower than the values of the wires that were made from Alloy #3 (625 MPa). Analysis of the data that are presented in Figure 8 shows the curves hardness H_v_–ultimate strength σ_b_ for the non-annealed wires to have an almost linear character; the mean value of the proportionality coefficient of H_v_/σ_b_ ~ 2.

**Figure 9:**
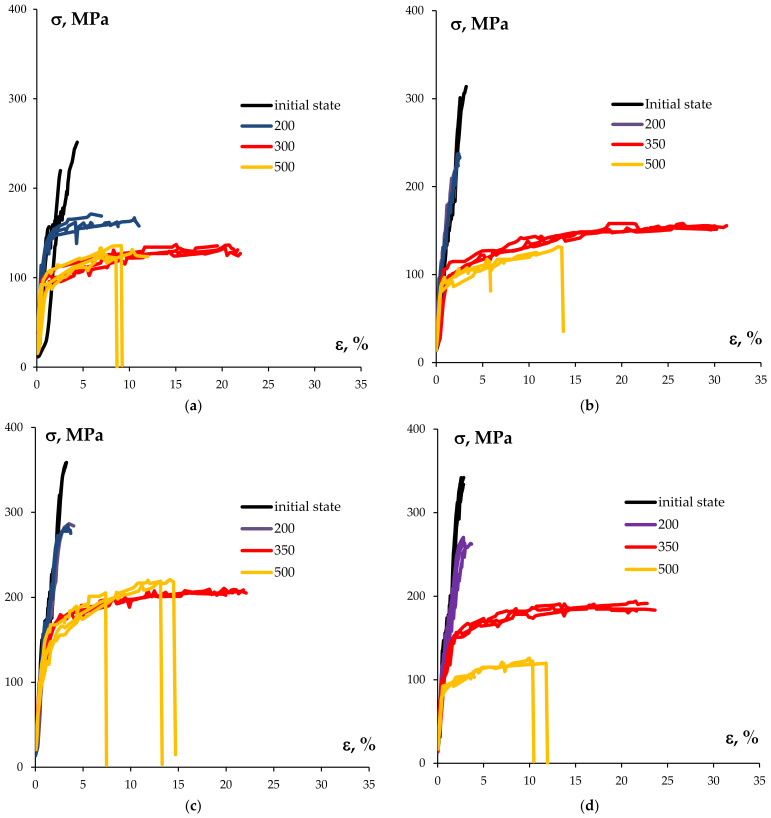

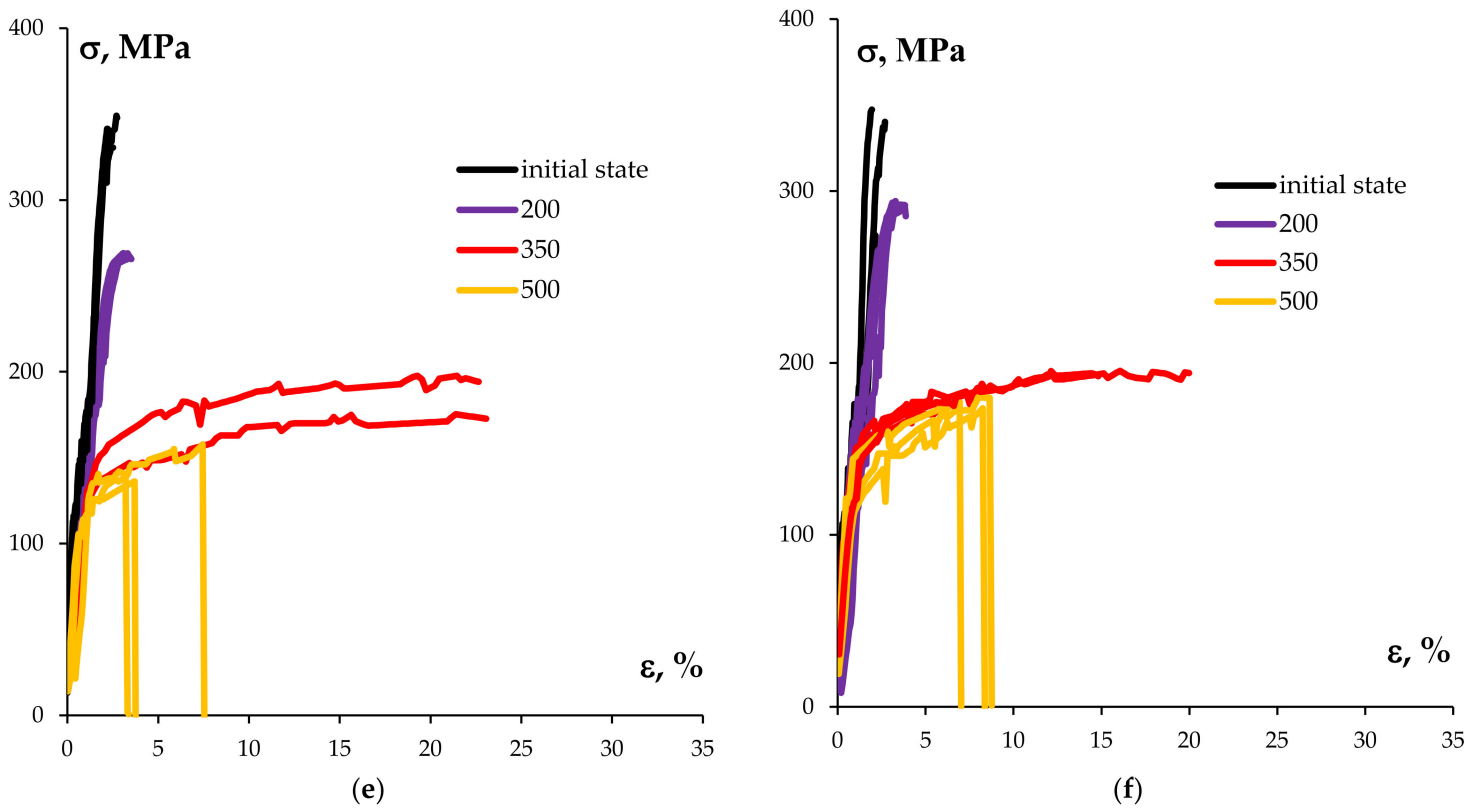
The tension curves σ(ε) of the sample wires made from alloys #1 (**a**), #2 (**b**), #3 (**c**), #4 (**d**), #5 (**e**), and #6 (**f**).

The tension curves σ(ε) for the sample wires in the initial state and after annealing at 500 °C (30 min) are presented in Figure 9. The results of the mechanical tests demonstrated the bimetallic wires to have a high strength in the initial state. The maximum values of the ultimate strength (σ_b_ = 370 MPa) were observed for the wires that were made from alloys #4–5 whereas the minimum ones were σ_b_ = 230 MPa for the wires that were made from pure Al (Alloy #1). The ultimate strength of the wire that was made from Alloy #3 containing 0.1%Sc was 345 MPa and, taking into account the scatter of the properties, appeared to be close enough to the ultimate strength of the wires that were made from alloys #4 and 5. The analysis of the tension curves σ(ε) shows the plasticity of all the wires in the initial state to be very small: the stable plastic flow stages were almost absent and the wires were brittle and broke upon achieving the ultimate strength (Figure 9). The relative elongation to failure (δ) didn’t exceed 0.5–1%.

**Figure 10:**
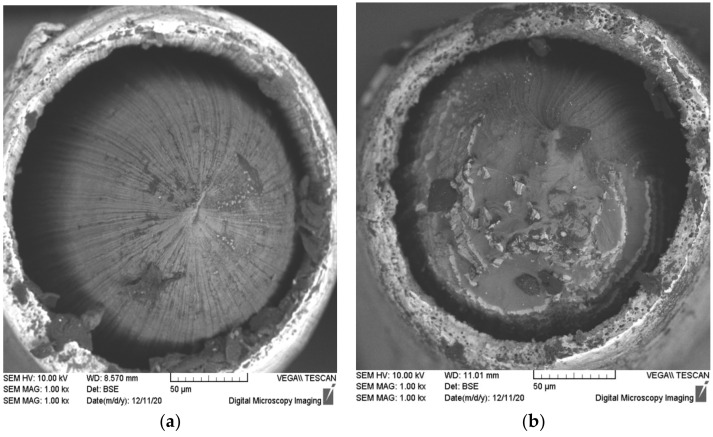

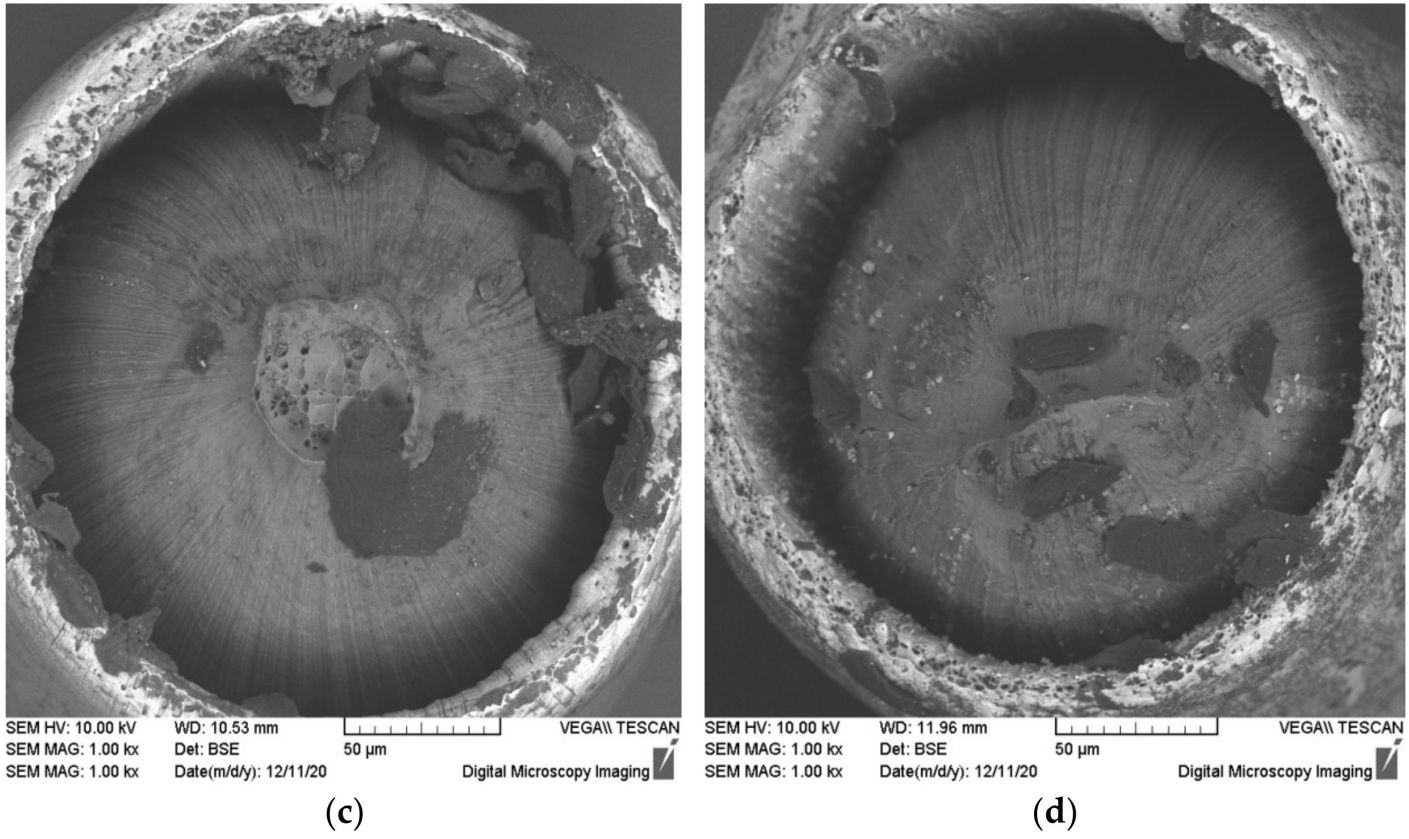
Fractographic analysis of the fractures of the sample bimetallic wires from Alloy #1 (**a**,**b**) and from Alloy #3 (**c**,**d**) after the tension tests: (**a**,**c**) without annealing; (**b**,**d**) and annealed at 500 °C for 30 min.

The fractographic analysis of the destruction areas (Figure 10) shows the destruction to become brittle after cutting, which goes through a shear along the slip plane. The detachment of the Cu cladding from the Al wire was observed on the fractures that may be evidence of an insufficient level of the adhesion strength of the Al–Cu interphase boundary. The Cu cladding collapsed viscously in the tension tests with the formation of micropores. There were no essential differences that were observed in the fractures of sample wires that were made from different alloys.

**Figure 11:**
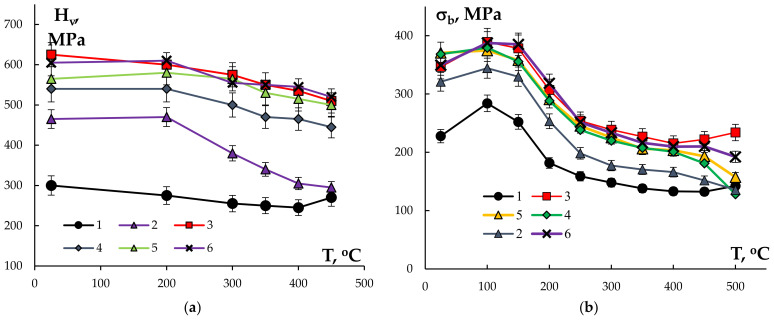

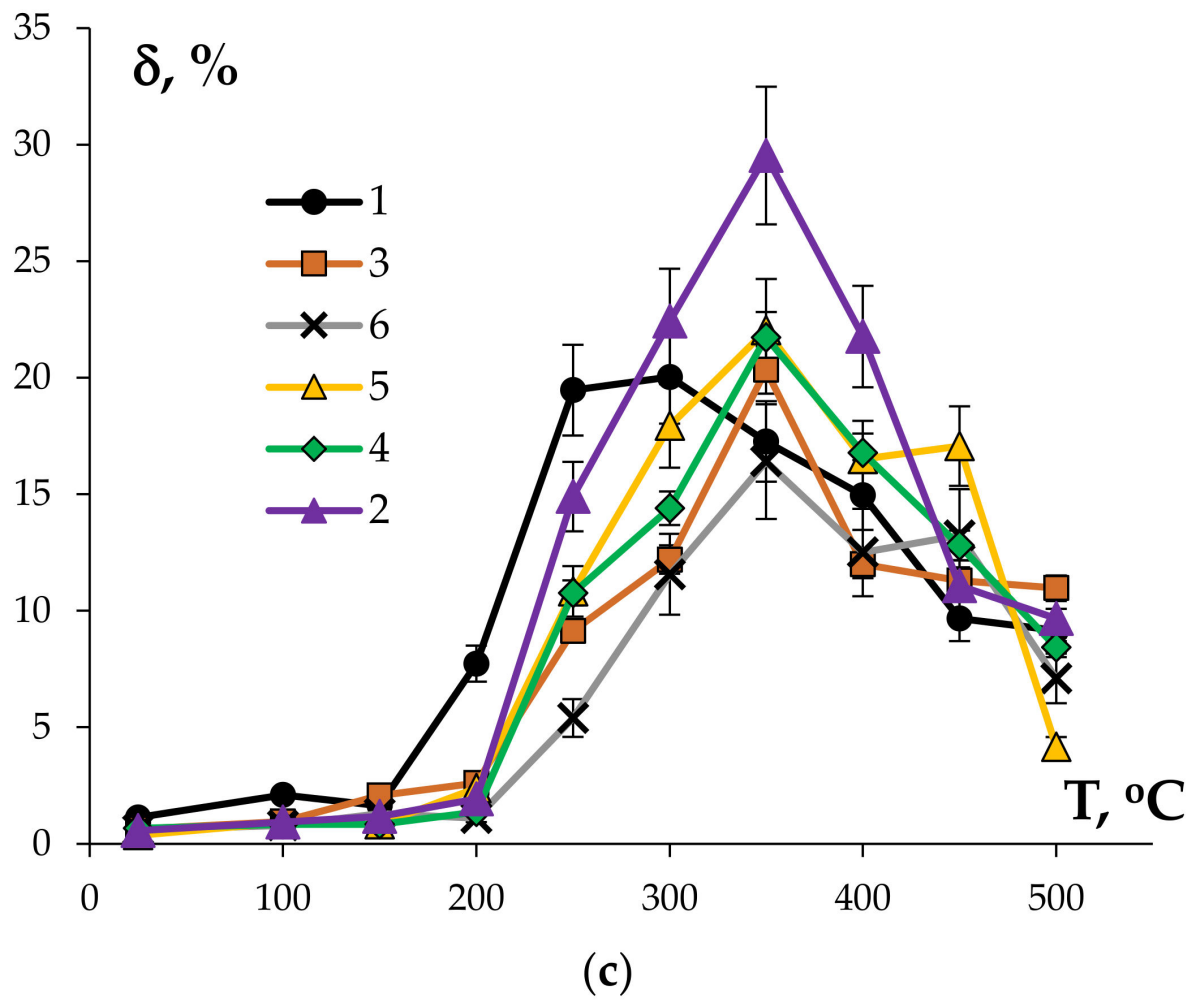
The dependencies of the mechanical properties on the 30 min annealing temperature for the sample bimetallic wires: (**a**) microhardness (H_v_); (**b**) ultimate strength (σ_b_); and (**c**) elongation to failure (δ).

Figure 11a shows the dependencies of the microhardness on the 30 min annealing temperature, which had a monotonously decreasing character. The microstructure measurements were performed in the central parts of the wire cross-sections. Note that despite a high microhardness of the wires that were made from Alloy #2 (Al-0.25%Zr) in the initial state, heating up to 200 °C and higher resulted in a rapid softening of the wires. After annealing at 450 °C, the microhardness of the wires (295 MPa) differed from the wires that were made from pure Al (270 MPa). The analysis of the dependencies H_v_(T) demonstrated the adding of Sc and Hf to the Al-0.25%Zr alloys decreased the intensity of decreasing of hardness during annealing. The highest microhardness (500–520 MPa) after annealing was observed for alloys #3, 5, and 6 containing from 0.05% up to 0.1% Sc. The microhardness of the annealed Alloy #4 containing 0.1%Hf (445 MPa) appeared to be slightly lower than the Sc-containing alloys #3 and #5 after similar annealing. The differences in the microhardness of the annealed alloys #2–4 were insufficient and didn’t exceed the scatter of properties.

The tension mechanical tests of the wires have shown the annealing to result in a reduction of the strength and in a considerable increasing of plasticity (Figure 11b,c). As one can see in Figure 9, the stage of stable plastic flow of the annealed wire metal has been observed in the σ(ε) curves while the magnitude of the elongation to failure (δ) for the majority of the wires reached the magnitude of more than 10%.

It is interesting to note that the dependencies δ(T) of all the wires were non-monotonous with maxima at 300 °C for the wire from Alloy #1 and 350 °C for the ones that were made from alloys #2–6. The maximum elongation to failure (~30 ± 2.1%) was achieved at the annealing of the wire that was made from Alloy #2 and the minimum of ~16 ± 1.7% at the annealing of Alloy #6. The annealing at temperatures over 350 °C resulted in a drastic reduction of the elongation to failure down to δ ~ 5–12%. Note that the reduction of plasticity was observed for the sample wires that were made from pure Al (Alloy #1) as well–δ ~ 9 ± 1.2% after annealing at 500 °C. The elongation of the wire from pure Al after annealing at 300 °C was ~20 ± 2.6%.

The maximum values of the ultimate strength (Figure 11b) were observed for the annealed wires that were made from alloys #3 and #6 containing 0.1% Sc. The magnitudes of the ultimate strength of the wires that were made from alloys #5 (Al-0.25%Zr-0.05%Sc-0.05%Hf) and #4 (Al-0.25% Zr-0.1%Hf) after annealing at 500 °C (30 min) were 195 MPa and 180 MPa, respectively. The magnitude of σ_b_ for the wire that was made from Alloy #2 after annealing at 500 °C (30 min) was close to that of the annealed wire from high-purity Al (see Table 1). Note also that the magnitudes of the aspect ratio H_v_/σ_b_ for the annealed wires were slightly higher than for the bimetallic wires in the initial state (Figure 8).

**Figure 12:**
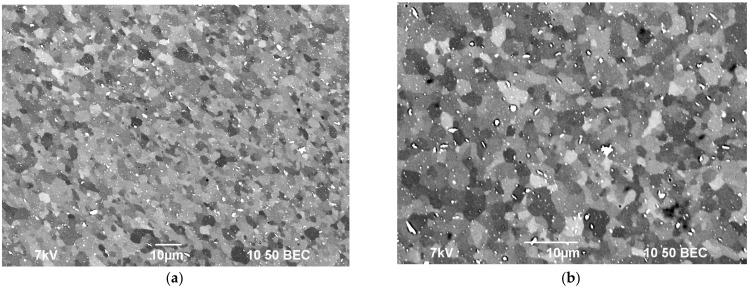
The microstructure of the central part of the wire that was made from Alloy #6 after annealing at 450 °C for 30 min. (**a**) small zooming; (**b**) large zooming.

An increased plasticity of the bimetallic wires evidences that the annealing resulted in the grain growth and in the formation of a uniform fine-grained structure whereas the increased hardness and strength of the sample wires is provided by the nucleation of the Al_3_(Zr,Sc,Hf) particles. Figure 12 shows the SEM images of the microstructure of an annealed bimetallic wire that was made from Alloy #6.

One can see from Figure 12 that a uniform, fine-grained microstructure was formed in the wires that were made from alloys #2–6 after the high-temperature annealing at 450 °C. The mean grain sizes were 3–5 μm or less.

**Figure 13:**
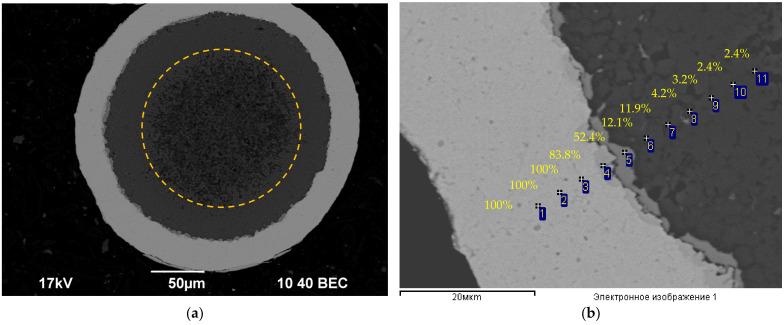
Macro- (**a**) and micro-structure (**b**) of a cross-section of the wire that was made from Alloy #1 and annealed at 500 °C for 30 min. The numbers in Figure 13b denote the Cu concentration (in wt.%) according to the EDS microanalysis (Al–balance).

The fractographic analysis of the fractures has shown the annealing not to affect the character of destruction essentially, but to result in an increasing of the destruction zone and in a decreasing of the degree of the detachment of the Cu claddings from the Al wires (Figure 11c,d). This may be evidence of a diffusion of Cu inside the Al wire surfaces to occur in the course of heating. This conclusion was confirmed indirectly by the results of the metallographic investigations of the macrostructure of the wire cross-sections. As one can see in Figure 13a, brighter-looking zones of ~30–40 μm in width were formed at the sides of the wire cross-sections after annealing. According to the EDS microanalysis results (Figure 13b), an increased Cu concentration (2–2.4 wt.%) was observed in these zones. The boundary of such a zone is marked by a yellow dashed line in Figure 13a. The results of the SEM investigations evidence brittle intermetallides to form at the interphase boundary of the Cu cladding with the Al wire after annealing (Point #5 in Figure 13b), which are, in our opinion, the origin of the reduced adhesion strength of the Al-Cu interphase boundaries. The precipitation that formed at the interphase boundary of the Cu-Al layers are presented in [36,37,38].

**Figure 14:**
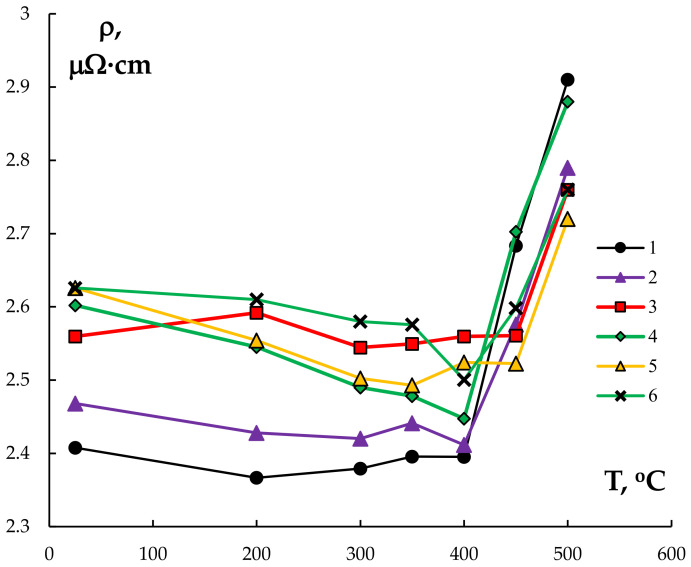
The dependencies of SER of the bimetallic wires on the 30 min annealing temperature.

Figure 14 presents the dependence of SER on the annealing temperature for the bimetallic wires of different compositions. First, note that the low SER values of the wires in the initial state likely originate from the presence of thin Cu claddings, the conductivity of which is higher than the one of Al alloys. This conclusion was confirmed by the results of SER measurements of the wires from pure Al (Alloy #1) without the Cu claddings–the measured SER values were 2.60–2.63 μΩ·cm. These values were close to the SER of Alloy #1 after casting as well as to the one after ECAP and annealing (see Section 3.1) within the measurement uncertainty (±0.03 μΩ·cm).

The SER magnitude for the bimetallic wire that was made from pure Al (Alloy #1) almost didn’t change with increasing annealing temperature up to 400 °C and was ~2.4 μΩ·cm (Figure 14). Further increasing the annealing temperature up to 450–500 °C resulted in a drastic increase of SER up to ~2.7–2.9 μΩ·cm that was probably due to an intensive diffusion of Cu into the Al wire and to the formation of a Al-Cu alloy in the subsurface layers of the wire (see Figure 13b). A similar effect was observed for the alloy Al-0.25%Zr (Alloy #2), where the nucleation of Al_3_Zr particles at these temperatures (below 400 °C) and annealing time (30 min) was very low intensive in (see [30,33,34]).

Note that a reduction of the elongation to failure of the bimetallic wires was observed after annealing at 400 °C (Figure 11c). This suggests that just the diffusion of Cu inside the Al wires and the formation of large particles at the interphase boundary (Figure 13b) are the origins of the reduction of plasticity of the bimetallic wires.

**Figure 15:**
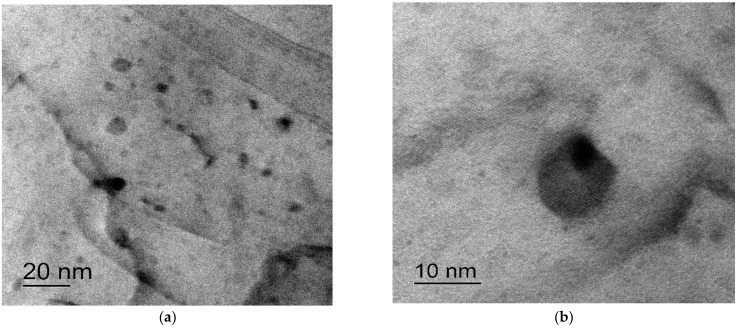
Nucleation of the Al_3_Zr intermetallide nanoparticles in the annealed wire made from Alloy #2. TEM. (**a**) small zooming; (**b**) large zooming.

One can see from Figure 14 that increasing the temperature of the 30 min annealing of the wires that were made from the Al-(0.2,0.25)%Zr-(Sc,Hf) alloys up to 350–400 °C resulted in a monotonous decreasing of the SER. According to [39], the decreasing of SER is caused by the alloy decomposition. This conclusion was confirmed by the results of the TEM investigations. As one can see in Figure 15, there are some intermetallide nanoparticles in the annealed sample wire material. The most expressed decreasing of the SER and, hence, the most intensive nucleation of the intermetallide nanoparticles was observed for the wires that were made from alloys #4 and #5.

## 4. Discussion

### 4.1. Effect of Annealing on the Microstrucutre Stability of the Al Alloys

As it has been mentioned above in Materials and Methods section, the nucleation of the Al_3_X intermetallide nanoparticles ensuring high thermal stability of the fine-grained microstructure of the bimetallic wires was one of the purposes of the preliminary annealing of the deformed workpieces at 300–400 °C.

According to [40], the scale of decreasing of the alloy SER (Δρ) in the course of annealing is proportional to the degree of the decomposition of solid solution ΔC and to the volume fraction of the nucleated particles f_v_. Here ΔC is the variation of the doping element concentrations in the solid solution during annealing. It follows from the data that are presented in Section 3.1 that the magnitudes of ΔC after annealing at 400 °C (2 h) were 0.51, 0.51, and 0.41 μΩ·cm for alloys #3, 4, and 5, respectively. Taking the SER of pure Al (Alloy #1) to be ρ_Al_ = 2.65–2.66 μΩ·cm (see Section 3.1), one can estimate the alloy magnitude ΔC according to the formula: ΔC_i_ = 100%·[Δρ/Δρ_max_] where Δρ_max_ = ρ_I_ − ρ_Al_ and ρ_i_ is the SER of the i^th^ alloy. For alloys of i = 3, 4, and 5, one obtains ΔC_i_ to be 65, 63, and 58%, respectively (Table 3). So far, the maximum alloy decomposition solid solution degree (ΔC_3_ ~ 65%) after annealing at 400 °C was observed for Alloy #3 (Al-0.25%Zr-0.1%Sc). Also, the minimum mean grain size (~150 μm) after annealing was observed in this alloy. Note that a complete substitution of 0.1%Sc (Alloy #3) for 0.1%Hf (Alloy #4) appeared to be very efficient; the mean grain size in Alloy #4 was ~200 μm (that was close to the mean grain size in annealed Alloy #4) at comparable alloy decomposition solid solution degree (ΔC_5_ ~ 58%). The maximum volume fractions of the second phase particles f_v_^max^ in the investigated Al alloys were calculated according to procedure that was described in [41]. The volume fractions of the particles that were nucleated in the course of annealing were calculated according to the formula: f_v_ = f_v_^max^·ΔC_i_. The values of f_v_^max^ and f_v_ for each alloy are presented in Table 3.

The partial substitution of Sc for Hf (alloys #5 and 6) was not efficient. The mean grain sizes in the annealed alloys #5 and 6 were 1 mm at high enough alloy decomposition solid solution degree (ΔC ~ 61–63%) and a considerable volume fraction of the nucleated particles (f_v_ ~ 0.27–0.34%). The origin of this is not clear enough yet.

We suggest this to be related to a separate nucleation of the Al_3_(Zr,Sc) and Al_3_(Sc,Hf) particles in the multicomponent Al alloys #5 and 6 containing Sc, Zr, and Hf simultaneously.

This suggestion is supported indirectly by the fact that the mean grain sizes in the alloys of the Al-Zr-Sc-Hf system (alloys #5 and 6) after annealing at 400 °C for 2 h appeared larger essentially than the ones in the alloys of the Al-0.25%Zr-0.1%Sc composition (~150–200 μm) and in the Al-0.25%Zr-0.1%Hf one (~200 μm). In our opinion, this means that the additional alloying of the Sc-containing Al alloys by Hf leads to formation of the incoherent Al_3_(Sc,Hf) particles during annealing (see [42,43]). The fast growth of the Al_3_(Sc,Hf) non-coherent particles will lead to an intensive grain boundary migration and, as a consequence, to the formation of more coarse-grained microstructure in the course of annealing that was observed in the experiment. So far, the stabilization of the nonequilibrium microstructure in Alloy #5 takes place preferentially by the nucleation of coherent Al_3_(Sc,Zr) nanoparticles, the volume fraction of which in Alloy #5 will be lower than the one in Alloy #3.

Also, it is interesting to note that wires #5 and 6 containing Sc, Zr, and Hf together manifested a lower elongation to failure after annealing than the wires from Al-0.25%Zr (Alloy #2), Al-0.25%Zr-0.10%Sc (Alloy #3), or Al-0.25%Zr-0.1%Hf (Alloy #4). This may indirectly prove that the non-coherent particles nucleating in alloys #5 and 6 have an increased energy of the “Al matrix–L1_2_ particle” interphase boundary or a higher shear modulus. These factors may impede “cutting” of the nucleated nanoparticles by lattice dislocations that lead to the formation of the disclination-type loops at the particles and to enhancement of the long-range internal strain fields (see [44]).

The mean grain sizes in the annealed Al-0.25%Zr-0.1%Sc alloy appeared to be 200/150 ~1.3 times smaller than the one in the Al-0.25%Zr-0.1%Hf alloy after similar annealing (see Section 3.1). The ratio of the volume fractions of the nucleated particles in these alloys was f_v2_/f_v5_ = 0.35/0.21 ~ 1.5 (see Table 3). The result that was obtained means that the insufficient increasing of the mean grain sizes observed in Alloy #4 as compared to Alloy #3 agrees with the decreasing of the volume fraction f_v_ according to Zener ratio: d_Z_ = k·R/f_v_ [45]. Here, k is a numerical factor depending on the particle geometry and on the interphase boundary energy and R is the mean size of the nucleated particles [45]. Note also that the volume fraction of the nucleated particles in the Al-0.25%Zr-0.1%Sc alloy appeared to be greater than the one in the Al-0.25%Zr-0.1%Hf alloy after similar annealing. It means that the formation of the Al_3_(Zr,Hf) particles goes slower than the Al_3_(Zr,Sc) ones but faster than the Al_3_Zr particles in Alloy #2 (Table 2).

Also, it is important to take into account that the Hf content (%) in Alloy #4 appeared to be lower than the Sc one (%) in Alloy #3 (Table 2). So far, the replacement of 0.1 wt.%Sc by 0.1 wt.%Hf will lead to a reduction of the maximum volume fraction of the Al_3_X particles, which may nucleate in the alloy at the same annealing conditions.

### 4.2. Effect of Annealing on the Stability of the Mechanical Properties of the Wires

The presence of the Cu claddings may affect the deformation behavior of the Al wires. First, it is related to the contributions of the deformation and destruction of the Cu claddings as well as of the destruction of the interphase boundary of the Cu cladding with the Al wire into the deformation behavior of the wires during the tension tests. Second, the intensive diffusion of Cu into the Al alloy may affect the tension test results indirectly.

In our opinion, the intensive Cu diffusion into the Al wire surfaces is one of the origins of nonmonotonous dependence δ(T) (see Figure 13b). The increase of the plasticity at the annealing temperatures <350 °C is a consequence of the recovery and recrystallization that leads to a decrease of the internal long-range stresses. The annealing at the temperatures over 350–400 °C leads to an intensive Cu diffusion into the Al wire surfaces and to the formation of large brittle Al-Cu intermetallides at the interphase boundaries between Cu and Al (Figure 13b). In our opinion, these processes lead to reduction of the relative elongation to failure of the bimetallic wires. The nucleation of the Al_3_(Zr,X) particles is an additional factor reducing the plasticity of the wires at elevated annealing temperatures. The formation of the Al_3_(Zr,X) blocks the dislocation slip in the crystal lattice and leads to the reduction of the plasticity of the Al wires.

One should pay special attention to the abnormally high intensity of diffusion of Cu into the Al wire surfaces. According to [46], the activation energy of Cu diffusion in Al crystal lattice (Q_v_) scatters from 15.6 to 16.9 kT_m_ whereas the pre-exponential factor is close to D_v0_ ~ 10^−6^ m^2^/s. Here T_m_ is the Al melting point and k is Boltzmann constant. Using the ordinary rule to calculate the diffusion mass transfer time τ_diff_ = x^2^/D_v_, one can show that at τ_diff_ = 30 min, D_v_ = D_v0_exp(−Q_v_/kT), and T = 450–500 °C (723–773 K) means that the expected characteristic scale of Cu diffusion in Al is x ~ 4–6 μm. This value is much less than the scale of the Cu diffusion into the Al wire surface that was observed experimentally (~30–40 μm, see Figure 13). In our opinion, the formation of the uniform fine-grained structure in the Al alloy in the course of rolling is one of main origins of deep penetration of Cu. It leads to preferential diffusion mass transfer along the grain boundaries, the diffusion permeability of which are known to exceed the one of the metal crystal lattice [47]. Since the intensity of grain boundary migration in alloys with different compositions will be different, one can expect the surface layers of the Al alloys to contain different Cu concentrations. According to the model of effect of small additives of the alloying elements on the diffusion permeability of the grain boundaries [48] and on the recrystallization temperature [49], it will also lead to change of the grain boundaries migration intensity in the surface layers of the Al wires. Different degrees of saturation with Cu for the surface layers of the wires that were made from different alloys is probably one of the origins of the observed different scales of the SER increase at the increased temperatures of annealing of the wires that were made from alloys with different compositions (Figure 14).

In this connection, to simplify the analysis of the results that were obtained, let us consider the effect of annealing on the microhardness of the central parts of the Al wires. In Figure 13a, this area is marked by the yellow dashed line.

**Figure 16:**
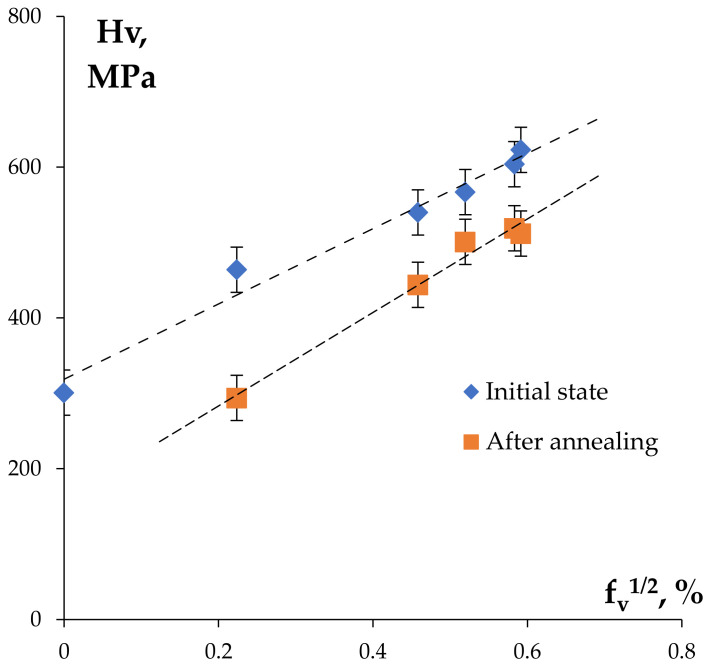
Dependencies of the microhardness of the Al wires H_v_ on the volume fraction of the nucleated particles f_v_.

Figure 16 presents the dependence of the microhardness on the volume fraction of the Al_3_X particles in the Al wires in the initial state and after annealing at 400–450 °C for 30 min. The volume fractions of the nucleated particles for the wires in the initial state were taken to be equal to the values of f_v_ in the same annealed Al alloys, which these wires have been fabricated from (Table 3). The value of f_v_ for each annealed wire was calculated as a sum of the volume fraction of the particles that were nucleated in the non-annealed wire and of the particles that were nucleated when annealing the bimetallic wire Δf_v_. The magnitude of Δf_v_ has been accepted to be proportional to the magnitude of decreasing of the SER as a result of the annealing of the wire (Δρ_2_) when heating this one up to 400 °C (see Figure 14, for the description of the procedure of calculation of the volume fraction–see Section 4.1).

Analysis of the results that were obtained showed that the curve H_v_(f_v_) that was plotted in the H_v_–$\sqrt{f_{v}}$ axes can be interpolated by a straight line with a good precision. This means that the dependence of the hardness of the central parts of the Al wires on the volume fraction of the second phase particles Al_3_X in the temperature interval up to 400 °C can be described using the Orowan equation: $H_{v}=\alpha \mathrm{Gb}\sqrt{f_{v}}/R$ where α is a numerical parameter, G is the shear modulus, and b is the Burgers vector [45].

## 5. Conclusions

The features of changes of the mechanical properties of the bimetallic microdoped Al wires with small cross-sections (∅0.26 mm) with high-purity Cu coatings upon annealing have been investigated.

Preliminary annealing of the Al alloys providing the nucleating of the second phase particles Al_3_(Zr,Sc,Hf) prior to making the wires has been shown to allow the preservation of the uniform fine-grained structure with the mean grain sizes of 3–5 μm as well as with an increased strength and hardness.

The samples that were made from the Al-0.25%Zr-(Sc,Hf) alloys were found to have the highest thermal stability. The wires made from the Al-0.25%Zr-(Sc,Hf) alloys had the highest hardness and ultimate strength after annealing. The character of fractures of the sample bimetallic composite wires in the tension tests was found to change upon annealing that was related to by the interdiffusion of Cu into the wire surfaces and of Al into the Cu cladding.

## Figures and Tables

**Table 1:** The composition and mechanical properties of conductor Al alloys.

Alloy	Alloying Elements Contents in the Alloy, wt.%			Mechanical Properties of the Wire			
				Initial State		After Annealing at 450 °C, 30 min	
	Zr	Sc	Hf	H_v_, MPa	σ_b_, MPa	H_v_, MPa	σ_b_, MPa
#1	–	–	–	300	230	270	135
#2	0.25	–	–	465	320	295	150
#3	0.25	0.10	–	625	345	510	235
#4	0.25	–	0.10	540	370	445	180
#5	0.25	0.05	0.05	565	370	500	195
#6	0.20	0.10	0.10	605	350	520	210

**Table 2:** The preparation regimes of the alloys.

Parameters	Alloy #1	Alloy #2	Alloy #3	Alloy #4	Alloy #5	Alloy #6
Preparation of the cast alloys						
Mold, mm	22 × 22 × 160, graphite					
Crucible 150 cm^3^	SiO_2_ + ZrO_2_					
Ar purge prior to casting, cycles	3					
Ar purge during heating, cycles	3					
Melt mixing	Induction casting					
Heating power, kW	4.5					
Time until melting of the components, s	520	510	515	510	515	480
Melt holding temperature, °C	780	810	820	820	820	820
Holding before pouring, min	3	9	20	20	20	20
Pouring temperature, °C	760					
Cooling time, s	50	40	250	250	250	250
incl. vibration time, s	50					
Deformation processing of the cast workpieces and wire making						
Deformation processing of the cast workpieces	ECAP (N_ECAP_ = 4, T_ECAP_ = 250 °C)					
Temperature of 2-h annealing, °C	300	300	400	400	400	400
Workpiece processing	Milling, scalping					
Wire making	Rolling in rolls at room temperature					

**Table 3:** Analysis of the parameters of the particles that were nucleating in the investigated alloys.

Alloy	Alloying Elements Concentration							f_v_^max^, %	ΔC_i_, %	f_v_, %
	Zr		Sc		Hf		Zr + Sc + Hf, at.%			
	wt.%	at.%	wt.%	at.%	wt.%	at.%				
#2	0.25	0.073	–	–	–	–	0.073	0.30	18	0.05
#3	0.25	0.073	0.10	0.06	–	–	0.133	0.54	65	0.35
#4	0.25	0.073	–	–	0.10	0.015	0.088	0.36	58	0.21
#5	0.25	0.073	0.05	0.03	0.05	0.008	0.111	0.45	61	0.27
#6	0.20	0.059	0.10	0.06	0.10	0.015	0.134	0.54	63	0.34

## Data Availability

Data is contained within the article.
